# Open-heart surgery using a centrifugal pump: a case of hereditary spherocytosis

**DOI:** 10.1186/s13019-016-0534-8

**Published:** 2016-08-26

**Authors:** Yuichi Matsuzaki, Hideyuki Tomioka, Masaki Saso, Takashi Azuma, Satoshi Saito, Shigeyuki Aomi, Kenji Yamazaki

**Affiliations:** The Heart Institute Japan, Department of Cardiovascular Surgery, Tokyo Women’s Medical University, 8-1 Kawada-cho, Shinjyuku-ku, Tokyo, 162-8666 Japan

**Keywords:** Hereditary spherocytosis, Adult cardiac, Bicuspid aortic valve, Cardiopulmonary bypass, Haptoglobin, Centrifugal pump

## Abstract

**Background:**

Hereditary spherocytosis is a genetic, frequently familial hemolytic blood disease characterized by varying degrees of hemolytic anemia, splenomegaly, and jaundice. There are few reports on adult open-heart surgery for patients with hereditary spherocytosis.

**Case presentation:**

We report a rare case of an adult open-heart surgery associated with hereditary spherocytosis. A 63-year-old man was admitted for congestive heart failure due to bicuspid aortic valve, aortic valve regurgitation, and sinus of subaortic aneurysm. The family history, the microscopic findings of the blood smear, and the characteristic osmotic fragility confirmed the diagnosis of hereditary spherocytosis. Furthermore, splenectomy had not been undertaken preoperatively.

The patient underwent a successful operation by means of a centrifugal pump. Haptoglobin was used during the cardiopulmonary bypass, and a biological valve was selected to prevent hemolysis. No significant hemolysis occurred intraoperatively or postoperatively.

**Conclusion:**

There are no previous reports of patients with hereditary spherocytosis, and bicuspid aortic valve. We have successfully performed an adult open-heart surgery using a centrifugal pump in an adult patient suffering from hereditary spherocytosis and bicuspid aortic valve.

## Background

Hereditary spherocytosis (HS) is a genetic, frequently familial hemolytic blood disease characterized by varying degrees of hemolytic anemia, splenomegaly, and jaundice. The disease is associated with various defects in any of the number of the proteins responsible for maintaining the shape and flexibility of the red blood cell (RBC), resulting in osmotically fragile and characteristically spherical RBCs. Cardiopulmonary bypass (CPB) can exacerbate hemolysis and subsequent renal dysfunction. There are few reports on open-heart surgery for adult patients with HS.

This study reports the case of a 63-year-old man with HS who underwent aortic valve replacement, valval aneurysm patch closure, and ascending aorta replacement.

## Case presentation

The patient was a 63-year-old man with HS, idiopathic aortic bileaflet, and subaortic aneurysm (SAA) caused by infective endocarditis. The SAA was incidentally discovered during a clinical evaluation for aortic regurgitation (AR); this was due to idiopathic aortic bileaflet and infective endocarditis that were diagnosed 10 years ago. Because of the patient’s HS history and severe anemia, a cardiologist conducted the medical follow-up. The patient’s LV systolic function worsened gradually and he had frequent episodes of dyspnea. Moreover, transapical echocardiography showed a decrease in the AR grading. On admission, the hemoglobin level was 8.7 mg/dL, hematocrit was 23.8 %, platelet count was 18.2 × 10^4^/μL, blood urea nitrogen was 15.1 mg/dL, and creatinine was 0.93 mg/dL. The physical examination revealed an anemic and icteric conjunctiva, and his skin appeared jaundiced. The laboratory findings of the patient’s blood smear revealed a type 1 + polychromasia.

**Fig. 1:**
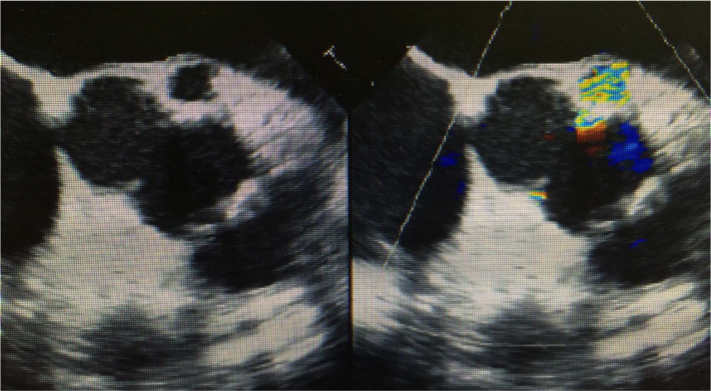
Subaortic aneurysm showed by TTE

The transthoracic echocardiogram showed a bicuspid aortic valve (adhesions on the left and right coronary cusps), a 10-mm SAA prolapsing at the atrio-ventricular continuity, an ascending aorta measuring 38 mm, and a grade IV central AR (Fig. 1). The left ventricular ejection fraction was preserved. The computer tomography angiography revealed a right coronary sinus aneurysm in the sagittal and axial plane.

A surgical intervention was indicated because of severe AR due to the bicuspid aortic valve, moderate dilation of ascending aorta, and SAA. The procedure was performed through a median sternotomy. A CPB was instituted by ascending aortic cannulation and bicaval drainage. Left atrial venting was carried out from the right upper pulmonary vein. Cardioplegia was performed by selective antegrade and retrograde perfusion with blood Frem’s solution and cold blood. Before initiating the CPB, we transfused four units of RBCs (hemoglobin of 12.1 mg/dL). Furthermore, human haptoglobin was also transfused.

**Fig. 2:**
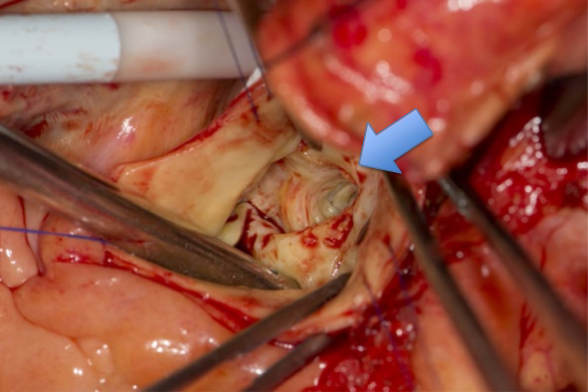
Subaortic aneurysm

**Fig. 3:**
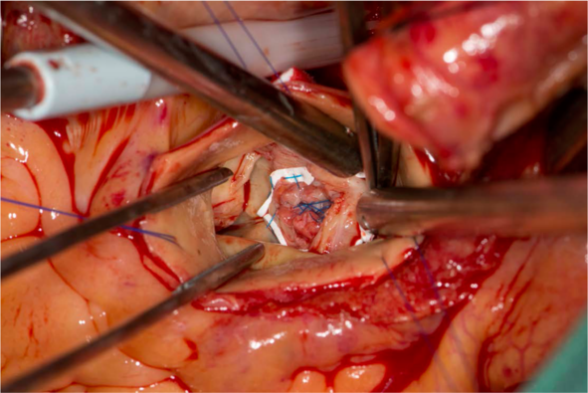
Repair of the Subaortic aneurysm (SAA)

At the time of the CPB, the centrifuge pump was selected rather than the roller pump, and perfusion index was 2.5 L/m^2^/min. First, the epicardial membrane was used for the repair of the SAA (Figs. 2 and 3). Second, aortic valve replacement was performed by using the biological valve (Magna EASE 21 mm, Carpentier-Edwards, Japan). Third, an ascending aorta replacement was performed under moderate hypothermia and cross-clamping condition. The aortic cross-clamp time was 182 min, and CPB time was 234 min. The urine remained clear throughout and after CPB, indicating that no significant hemolysis had occurred during the procedure. The patient was extubated on day 1, and all the vasoactive infusions were discontinued on day 3 postoperatively.

Despite the 1.5 L RBC transfusion during the operation, the immediate postoperative blood smear did not show any spherocytes and other abnormal RBC morphologies. Postoperative bleeding was minimal, and further RBC transfusions were performed on day 4 and 14 postoperatively.

The echocardiography showed no residual AR, and the patient was discharged from the hospital on day 18 without any hemolysis. At the 6-month follow-up, he was doing well. Recent echocardiography showed no residual AR, and good left ventricular function. Hematogenic investigation showed the hemoglobin level to be maintained at 10.6 g/dL without any transfusion. The total bilirubin and lactate dehydrogenase levels were 3.9 mg/dL, and 303 IU/L, respectively (Table 1).

**Table 1:** Changes in the laboratory data

	Before operation	Ope	Day 3	Day 10	Day 18	6 months
RBC (10^6^/μL))	243	339	265	276	362	295
Hb (g/dl)	8.7	10.5	7.9	8.2	11	10.6
Hct (%)	23.8	29.8	24.7	25.1	32.9	28.7
T.B. (mg/dL)	3.3	9.8	6.6	1.6	2	3.9
LDH (IU/L)	141	265	380	262	302	303
Haptoglobin	24	<10	<10	<10	<10	22

## Discussion

Hereditary spherocytosis, an autosomal dominant or recessive trait most commonly (though not exclusively) found in the Northern European and Japanese families, affects one in 2000 individuals [1].

There are few publications about the HS management during an open-heart surgery and there were no reports of patients with HS, bicuspid aortic valve, and SAA. A variety of approaches have been proposed for HS patients to avoid hemolysis during surgery. These include cardiac surgery without CPB, preemptive splenectomy [2], administration of haptoglobin to reduce plasma-free hemoglobin [3, 4], use of poloxamer 188 (a non-ionic antihemolytic detergent that protects RBC membrane during CPB) [5], or simply proceeding with surgery employing CPB without any special measures [5–7]. No significant hemolysis or renal failure has been reported in these instances and no trial has compared these various approaches.

It must be noted that in Japan, poloxamer 188 is not permitted for use on humans; therefore, we did not consider this when analyzing our case.

Although splenectomy is the only treatment for HS, we did not proceed because this patient had the high operative risk and he did not present with splenomegaly.

Our patient had multiple risks contraindicating cardiac surgery; he had a bicuspid aortic valve and an enlarged ascending aorta, which necessitated a valve change. In addition, he had SAA and his HB level was low (8.7 mg/dL); thus, a straightforward procedure was not possible. For all these reasons and for the clinical safety of the patient, we had to consider other alternatives. To reduce the risk of hemolysis during operation, we had four measures as follows.

First, RBC (HB of 10 mg/dL) transfusion was performed preoperatively. Second, aortic valve replacement was performed by using a biological valve, probably less hemolysis than mechanical valve. Third, we administered haptoglobin. Because haptoglobin can link to the free hemoglobin and change the complex form, this link can help the hepatic metabolism and prevent renal damage. In this case, although haptoglobin was transfused during and after the operation, haptoglobin level remained low. This could be explain by the fact that the speed of hemolysis for an HS patient is high, implying that the patient requires substantial amount of haptoglobin. Finally, we used a centrifugal pump because the risk of hemolysis is less with a centrifugal pump rather than with a roller pump [8]. The hematology-related approach, the improvement in surgical techniques, and the CPB technology will certainly reduce the complications associated with surgical intervention in patients with HS.

## Conclusion

The outcome in this case was good as evidenced by the no residual AR, lack of hemolysis, and the preserved splenic function. We can conclude that we have successfully performed an adult open-heart surgery by using a centrifugal pump in a patient suffering from HS and bicuspid aortic valve.

## Abbreviations

AR: Aortic regurgitation
CPB: Cardiopulmonary bypass
HS: Hereditary spherocytosis
RBC: Red blood cell
SAA: Subaortic aneurysm
